# Multiscale Entropy Feature Extraction Method of Running Power Equipment Sound

**DOI:** 10.3390/e22060685

**Published:** 2020-06-19

**Authors:** Yongjie Zhai, Xu Yang, Yani Peng, Xinying Wang, Kang Bai

**Affiliations:** 1 Department of Automation, North China Electricity Power University, Baoding 071003, China; zhaiyongjie@ncepu.edu.cn (Y.Z.); yxjkn7@ncepu.edu.cn (X.Y.); pengyani@ncepu.edu.cn (Y.P.); baikang_zdh@ncepu.edu.cn (K.B.); 2 Department of Computer, North China Electricity Power University, Baoding 071003, China

**Keywords:** running power equipment sound, feature extraction, improved complementary ensemble empirical mode decomposition with adaptive noise, multiscale improved permutation entropy

## Abstract

The equipment condition monitoring based on computer hearing is a new pattern recognition approach, and the system formed by it has the advantages of noncontact and strong early warning abilities. Extracting effective features from the sound data of the running power equipment help to improve the equipment monitoring accuracy. However, the sound of running equipment often has the characteristics of serious noise, non-linearity and instationary, which makes it difficult to extract features. To solve this problem, a feature extraction method based on the improved complementary ensemble empirical mode decomposition with adaptive noise (ICEEMDAN) and multiscale improved permutation entropy (MIPE) is proposed. Firstly, the ICEEMDAN is utilized to obtain a group of intrinsic mode functions (IMFs) from the sound of running power equipment. The noise IMFs are then identified and eliminated through mutual information (MI) and mean mutual information (meanMI) of IMFs. Next, the normalized mutual information (norMI) and MIPE are calculated respectively, and norMI is utilized to weigh the corresponding MIPE result. Finally, based on the separability criterion, the weighted MIPE results are feature-dimensionally reduced to obtain the multiscale entropy feature of the sound. The experimental results show that the classification accuracies of the method under the conditions of no noise and 5 dB reach 96.7% and 89.9%, respectively. In practice, the proposed method has higher reliability and stability for the sound feature extraction of the running power equipment.

## 1. Introduction

As the basic unit of a power system, the power equipment is of great importance and its operation status affects the safety, stability, and efficiency of the power grid. With the increase of power equipment, the possibility of equipment failure and the resulting loss rises accordingly. Therefore, it has become an important research issue to study safer and more effective state monitoring methods, so as to improve the monitoring sensitivity, realize fault warning, and improve the generalization of fault diagnosis systems [1,2,3].

In recent years, the state monitoring and fault diagnosis technology for power equipment with modern information technology has been vigorously promoted, and many kinds of equipment operation information have been applied to various fields [4]. The researchers studied different forms of equipment condition monitoring and fault warning from the aspects of equipment images [5,6,7,8], electromagnetic waves [9], ultrasonic waves [10,11], temperature [12,13], pressure [14] and vibration signals [15,16,17]. However, the development of equipment fault is a slow and nonlinear process, and the influencing factors are also multi-dimensional, complex, and changeable. In fact, these factors are not sensitive to the change of low load, making it a challenge to obtain timely and accurate early warning. Besides, the application of the information above often requires multiple sensors for contact measurement and the information collected is especially dependent on the location of the sensors, which brings great difficulties for equipment maintenance and adaption to the harsh environment and high load variation in the power plant. In view of this, many efforts have been made to get new detection information.

As another research field of artificial intelligence at present, computer hearing develops rapidly, but most of the progress focuses on the speech recognition technology, while the application in the power system has not been paid attention to [2,18]. The nonstationary noise generated by operation is the main information source of computer hearing in power equipment condition monitoring. The sound information has the advantages of wide sources, non-contact measurement, real-time monitoring, and strong fault warning ability, almost comparable to all current state detection parameters [19]. Moreover, the sound of running power equipment contains a lot of characteristic information, which helps to improve the detection and classification accuracy significantly. Therefore, it is extremely important to study the sound feature extraction technology for running power equipment [20,21].

Due to the coupling effect of operating noise between the power equipment, the acquired sound is often high noisy and nonlinear, making it difficult to extract effective features from such a complex signal. Many efforts had been done to study the mechanism of running equipment sound by simulation and experiment [22,23,24,25], frequency spectrum analysis [26,27,28], and wavelet analysis methods [29,30,31]. However, these methods are incapable of adaptively analyzing and eliminating the noise interference between the devices [32]. Hence, the empirical mode decomposition (EMD) [33] was utilized to remove the noise in the equipment sound. EMD, proposed by Huang, is an adaptive decomposition method for nonlinear and non-stationary signals. It is data-driven and parameter-free when decomposing a time series adaptively into a group of intrinsic mode functions (IMFs). The IMFs can be classified into noise IMFs and signal IMFs according to different center frequencies. However, a serious defect of the EMD is the mode mixing, which means that signals with different center frequencies will appear in the same mode. In order to overcome this problem, the ensemble empirical mode decomposition (EEMD) was proposed by Wu and Huang in 2009 [34]. The EEMD can effectively solve the mode mixing by adding white Gaussian noise, but it also generates two problems. Firstly, the reconstructed signal inevitably contains residual noise. Secondly, the different ways of adding noise to the signal will result in a different number of modes. In light of this, the complementary ensemble empirical mode decomposition with adaptive noise (CEEMDAN) was proposed [35]. Due to its powerful complex signal analysis capability, CEEMDAN is favored in different fields such as fault diagnosis [36,37,38], seismology [39], traffic flow prediction [40], and medicine [41,42]. However, CEEMDAN may produce some spurious modes in the early stage of decomposition [43]. Colominas et al. proposed an improved complementary ensemble empirical mode decomposition with adaptive noise (ICEEMDAN), which is an improved version of CEEMDAN [43]. ICEEMDAN calculates the local mean of the original signal to obtain each mode. By using ICEEMDAN, not only can the noise in the mode be reduced, but also the residual spurious modes problem caused by signal overlap can be reduced.

Entropy theory has shown a remarkable effect extracting the characteristic information of nonlinear and non-stationary signals in power equipment [38]. Various entropy algorithms have been put forward, including the sample entropy (SE) [44], fuzzy entropy (FE) [45], and permutation entropy (PE) [46]. Among them, PE becomes popular in the industrial field due to its convenient calculation and simple theory [47]. Nevertheless, there are two problems: (1) PE is sensitive to small changes in signal and susceptible to noise. (2) the entropy estimation results obtained by PE are liable to be affected by equalities in the signal. In order to solve the above problems, Chen Z et al. proposed the improved permutation entropy (IPE) [48]. By using a uniform quantification (UQ) operator to symbolize the signal, the algorithm not only improves the measurement accuracy of signal complexity but also makes noise interference more robust. The signals generated from complex systems usually exhibit multiscale structures [49], but the entropy algorithms above are all single-scale based. On account of this, Costa et al. [49] proposed a coarse-graining process, and it can be combined with an arbitrary entropy estimation algorithm for multiscale analysis. Combining the coarse-graining process with IPE, the multiscale improved permutation entropy (MIPE) can be obtained [48].

Since the mode decomposition-based and entropy-based techniques have a lot of advantages in processing complex time series, in this paper, a feature extraction method combining normalized mutual information (norMI) and separability criterion is proposed based on ICEEMDAN and MIPE to process the sound of running power equipment in this paper. The proposed method firstly eliminates the noise of the acoustic signals. Compared with the existing feature extraction technologies, it mines the multiscale characteristics of the sound based on ICEEMDAN and MIPE. Moreover, norMI is employed to weight MIPE results, which takes the importance of signal IMFs into account and makes use of the separability criterion to extract effective features and reduce dimensions from the weighted MIPE results. In such a way, the aim of revealing the inherent nonlinear feature of the sound without any noise interference can be achieved.

The rest of the paper is organized as follows: the proposed feature extraction theory is described in Section 2; the simulation and experimental results are provided in Section 3 and Section 4 respectively; and the paper is concluded in Section 5.

## 2. Algorithm

### 2.1. ICEEMDAN

**Figure 1:**
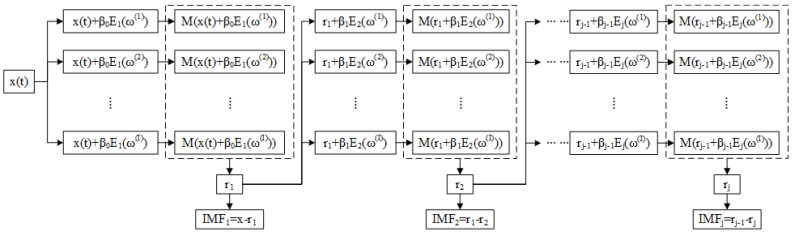
Flowchart of improved complementary ensemble empirical mode decomposition with adaptive noise (ICEEMDAN).

The flowchart of the ICEEMDAN algorithm is shown in Figure 1.

Given a composite signal x(t), *t* represents the sampling sequence of the composite signal. Let $E_{k}\left(\cdot \right)$ be the *k*th IMF obtained by EMD and define M(·) as the operator to calculate the local mean of a signal, and the ICEEMDAN algorithm is described as follows:

(1) Calculate $E_{1}\left(x\right)=x-M\left(x\right)$. For $x^{i}\left(t\right)=x\left(t\right)+\beta_{0}E_{1}\left(\omega^{\left(i\right)}\right),i=1,2,\ldots ,I$, the first residue is obtained by $r_{1}=\bar{M\left(x^{i}\left(t\right)\right)}$, where $\omega^{\left(i\right)}$ denotes ith added white Gaussian noise with zero mean and unit variance, *I* is the predefined ensemble sizes denoting the number of added $\omega^{\left(i\right)}$, and $\bar{M\left(x^{i}\left(t\right)\right)}$ denotes the action of averaging throughout the realizations of $M\left(x^{i}\left(t\right)\right),i=1,2,\ldots ,I$.
(2) The first mode can be written as ${\mathrm{IMF}}_{1}=x-r_{1}$.
(3) Calculated the second residue by $r_{2}=\bar{M\left(r_{1}+\beta_{1}E_{2}\left(\omega^{\left(i\right)}\right)\right)}$, and the second mode can be expressed as ${\mathrm{IMF}}_{2}=r_{1}-r_{2}$.
(4) For j=3,4,5,…,J, the *j*th residue and *j*th mode can be obtained by $r_{j}=\bar{M\left(r_{j-1}+\beta_{j-1}E_{j}\left(\omega^{\left(i\right)}\right)\right)}$ and ${\mathrm{IMF}}_{j}=r_{j-1}-r_{j}$ respectively, where *J* is the number of IMFs.
(5) Repeat Step (4) until all IMFs are obtained.

The constant $\beta_{j-1}$ is selected to adjust the signal-to-noise ratio (SNR) between the residual noise and the added noise. For j=1, $\beta_{0}=\varepsilon_{0}std\left(x\right)/std\left(E_{1}\left(\omega^{\left(i\right)}\right)\right)$, where std(·) represents the operator of standard deviation (SD) and $\varepsilon_{0}$ is the reciprocal of the expected SNR between the input signal x(t) and the first added noise [43]. For j≥2, $\beta_{j}=\varepsilon_{0}std\left(r_{j}\right)$.

### 2.2. Mutual Information, Mean Mutual Information and Normalized Mutual Information

The mutual information (MI) between two discrete random variables *X* and *Y* can be expressed as:

(1) $$\mathrm{MI}\left(X,Y\right)=\sum_{y\in Y}\sum_{x\in X}p\left(x,y\right)\log\left[\frac{p(x,y)}{p(x)p(y)}\right]$$

where p(x) and p(y) represent the probability density function of *X* and *Y* respectively, and p(x,y) denotes their joint probability density function. The mutual information is a measure of the correlation between two random variables [50]. If *X* and *Y* are independent, MI(X,Y)=0. Generally, the signal IMFs are more correlated with the original signal than the noise IMFs. Therefore, the mutual information can be used as an indicator to distinguish the signal IMFs from IMFs. The proposed method adopts the mean mutual information (meanMI) as a standard to compare the correlation between IMF and the original signal, which can be calculated as:

(2) $$\mathrm{meanMI}=\frac{1}{J}\sum_{j=1}^{J}\mathrm{MI}\left({\mathrm{IMF}}_{j},x\right)$$

where *J* denotes the number of IMFs decomposed from the original signal. If the MI of signal IMFs is larger than meanMI, it can be called a signal IMF.

In order to calculate the weighted MIPE (refer to Section 2.5), the norMI of each signal IMF is defined as:

(3) $${\mathrm{norMI}}_{i}=\frac{\mathrm{MI}\left({\mathrm{IMF}}_{i},x\right)}{\sum_{l}^{P}\mathrm{MI}\left({\mathrm{IMF}}_{l},x\right)},i=1,2,\cdots ,P$$

where *P* denotes the number of signal IMFs.

### 2.3. MIPE

Given a time series x(i),1≤i≤N and the time scale factor *s*, the subsequence $y_{i}^{s}$ can be calculated by the coarse-graining process, expressed as:

(4) $$y_{j}^{s}=\frac{1}{s}\sum_{i=(j-1)s+1}^{js}x\left(i\right),1\leq j\leq \frac{N}{s}$$

Given the embedding dimension *m* and the time delay *t*, the embedding vectors $z^{s}$ of $y_{i}^{s}$ are defined as:

(5) $$z^{s}=\left[y_{j}^{s},y_{j+t}^{s},\cdots ,y_{j+(m-1)t}^{s}\right],$$

where j=1,2,…,N/s−(m−1)t. Then, the embedded vector is symbolized based on the uniform quantization (UQ) operator in Reference [51]. The UQ operator can be represented as:

(6) $$\mathrm{UQ}\left(x\right)=\left\{\begin{array}{cc} 0 & x_{\min}\leq x\leq \Delta \\ 1 & \Delta \leq x\leq 2\Delta \\ \vdots & \vdots \\ L-1 & \left(L-1\right)\Delta \leq x\leq x_{\max} \end{array}\right.$$

where *x* is the input series, $x_{\min}$ and $x_{\max}$ the minimum and maximum values of *x* respectively, *L* is the predefined discretization level, and the procedure parameter $\Delta =(x_{\max}-x_{\min})/L$. Obviously, for the input series *x*, the UQ operator can produce an integer sequence ranging from 0 to L−1.

Based on Equation (6), the initial column vector of the embedded vector $z^{s}$ is symbolized to obtain the symbolized sequence $sym_{1}^{s}$, and then all column vectors of the embedded vector $z^{s}$ are symbolized based on the following equation:

(7) $$sym_{k}^{s}=sym_{1}^{s}+\frac{\left(z_{k}^{s}-z_{1}^{s}\right)}{\Delta },2\leq k\leq m$$

Next, similar to PE, each $sym_{l}^{s}\left(1\leq l\leq L^{m}\right)$ corresponds to the pattern $\pi_{l}$, and the probability distribution $p_{l}$ of $\pi_{l}$ can be calculated. Here $\pi_{l}$ denotes *l*th permutations of all permutations. These permutations are considered as the possible order types of *L* different numbers. The normalized IPE on scale factor *s* is written as:

(8) $$H_{IPE}^{s}\left(m,t,L,s\right)=\frac{\left[-\sum_{l=1}^{h}p_{l}^{s}\times \ln\left(p_{l}^{s}\right)\right]}{\ln\left(L^{m}\right)}$$

where $1\leq h\leq L^{m}$, and $\ln(L^{m})$ is the maximum value of $H_{IPE}^{s}$ which will be reached only when the patterns have a uniform distribution.

The MIPE vector can be computed by traversing all scale factors from 1 to *s*. The pseudo-code of MIPE is given in Algorithm 1.

**Algorithm 1:** Extract multiscale improved permutation entropy (MIPE) vector form time series.

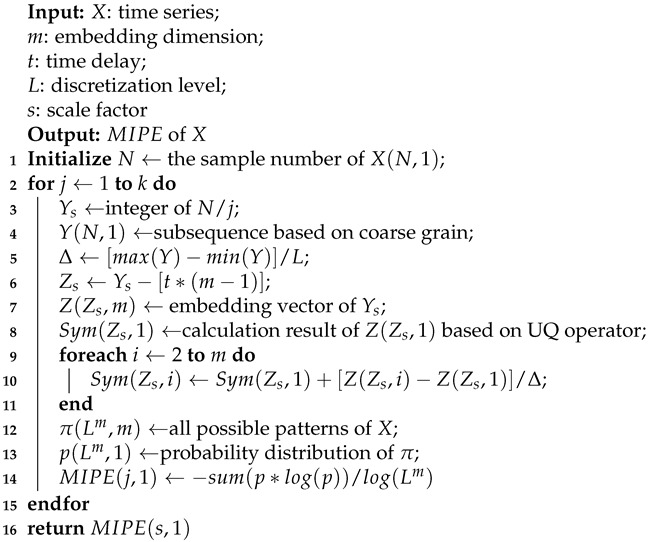



### 2.4. Separability Criterion

Suppose the finite feature vectors contain *N* samples belonging to *C* classes, and the sample number of the *i*-th class is $N_{i},1\leq i\leq C$. The interclass discrete matrix $S_{B}$ and the intra-class discrete matrix $S_{W}$ can be represented by Equations (9) and (10), respectively, and the separable criterion value (SC) can be expressed by Equation (11):

(9) $$S_{B}=\sum_{i=1}^{C}\frac{N_{i}}{N}\left(m_{i}-m\right){\left(m_{i}-m\right)}^{T}$$

(10) $$S_{W}=\sum_{i=1}^{C}\frac{N_{i}}{N}E_{i}$$

(11) $$SC=tr\left(S_{W}^{-1}\cdot S_{B}\right),$$

where $m_{i}$ is the mean value of the *i*-th-class sample feature vectors, *m* is the mean of all sample feature vectors, $E_{i}$ is the covariance matrix of the *i*-th-class sample feature vectors, and tr(·) is an operator to compute the trace of a matrix. $S_{B}$ and $S_{W}$ respectively represent the inter-class distance and intra-class distance of the sample feature vectors. The larger the inter-class distance and the smaller the intra-class distance, the larger SC is; that is, the classes are more separable. By quantifying the separability criterion, the effective information in the feature vectors can be effectively identified and reduced [52].

### 2.5. Proposed Feature Extraction Method

**Figure 2:**
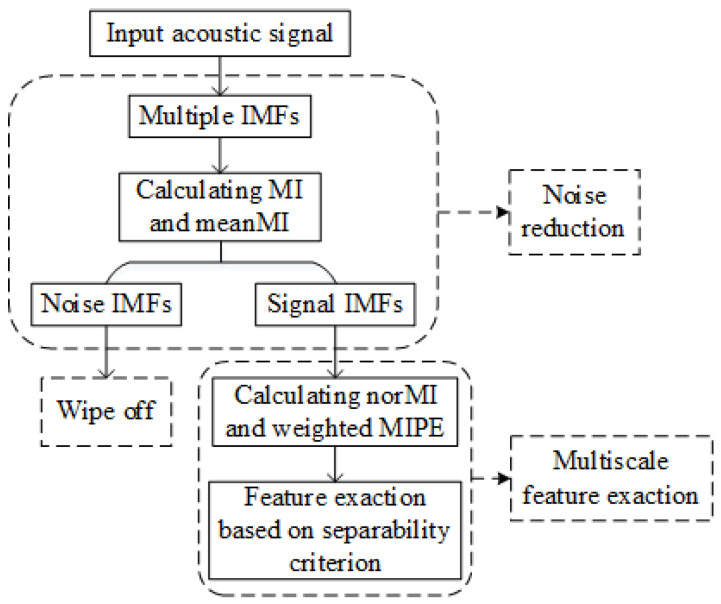
Flowchart of proposed feature extraction method.

The detailed flowchart of the proposed feature extraction method is shown in Figure 2. The specific steps of the algorithm are as follows:

(1) Decompose the input signal by the ICEEMDAN algorithm to obtain a group of IMFs.
(2) Calculate MI and meanMI of IMFs, and the signal IMFs can be identified by comparing MI of each IMF with meanMI (MI > meanMI).
(3) Calculate norMI and MIPE of each signal IMF, respectively.
(4) Use NorMI as the weight coefficient to weigh the corresponding MIPE results, and the weighted MIPE results are defined as the weighted sum.
(5) Extract the multiscale entropy feature vector by applying the separability criterion for dimension reduction to the weighted MIPE results.

## 3. Algorithm Simulation

### 3.1. Analysis on Artificial Signal Based on ICEEMDAN

In this section, the performance of ICEEMDAN on an artificial signal will be analyzed. The signal was generated by Equation (12), where the number of sampling points of each signal was 1000 (1≤n≤1000). $s_{1}\left(n\right)$ and $s_{2}\left(n\right)$ are the two source signals to be decomposed, $s_{3}\left(n\right)$ are the cosine noise, S(n) are the mixed artificial signals, and ω(n) denotes the white Gaussian noise with zero mean and unit variance.

(12) $$\left\{\begin{array}{c} s_{1}\left(n\right)=\left\{\begin{array}{cc} \sin(2\pi \cdot 0.20(n-201)) & 201\leq n\leq 350\\ \cos(2\pi \cdot 0.26(n-501)) & 501\leq n\leq 750\\ 0 & \;\mathrm{other}\; \end{array}\right.\\ s_{2}\left(n\right)=\sin\left(2\pi \cdot 0.05\left(n-1\right)\right)\\ s_{3}\left(n\right)=\cos\left(2\pi \cdot 0.5\left(n-1\right)\right)\\ S\left(n\right)=s_{1}\left(n\right)+s_{2}\left(n\right)+\sqrt{0.001}\times \left(s_{3}\left(n\right)+\omega \left(n\right)\right) \end{array}\right.$$

**Figure 3:**
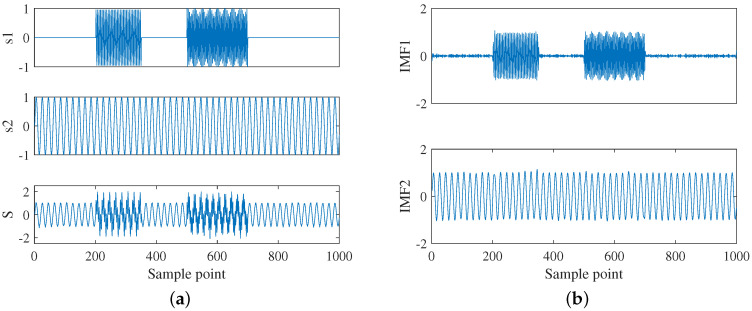

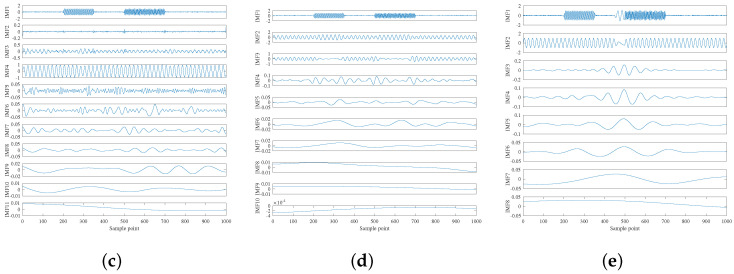
Artificial signal and decomposition results: (**a**) waveform of the artificial signal; (**b**) ICEEMDAN; (**c**) CEEMDAN; (**d**) EEMD; and (**e**) EMD.

The source signal waveform of the artificial signal is shown in Figure 3a. The analysis results of ICEEMDAN, CEEMDAN, EEMD, and EMD are shown in Figure 3b–e, respectively. To evaluate the algorithm, the added noise amplitude $\varepsilon_{0}$ and the ensemble sizes *I* were selected as 0.2 and 100, respectively; the maximum number of iterations was 10,000. The following studies will use the same parameters. Due to the limitations of EMD [34], it can be seen from Figure 3e that the mode mixing occurred in EMD. From IMF3 to IMF8, each IMF had no fixed central frequency. Comparing Figure 3d with Figure 3e, the mode mixing problem of EEMD is improved, but the mode mixing still exists at some signal mutation positions (*n* = 200, 350, 500, and 750). As can be seen from Figure 3c that although the IMFs (IMF1 and IMF4) obtained by CEEMDAN were similar to the source signal, it generated spurious modes such as IMF2 and IMF3. The decomposition results of ICEEMDAN were consistent with the actual situation, which shows the superiority of this algorithm. Therefore, ICEEMDAN is used to decompose the acoustic signal of the electric equipment in this paper.

### 3.2. Analysis on Chaotic Signals Based on MIPE

In order to verify the effectiveness of the method proposed in Section 2.3, the entropy feature extraction was carried out on four typical chaotic signals, the Lorenz chaotic system, namely the Rossler chaotic system, the Duffing chaotic system, and the Chen chaotic system. For the given appropriate parameters, these four systems have chaotic characteristics. The Lorenz system can be expressed as:

(13) $$\begin{array}{c} \dot{x}=-a\left(x-y\right)\\ \dot{y}=-xz+cx-y\\ \dot{z}=xy-bz \end{array},$$

where *a* = 10, *b* = 8/3, and *c* = 28.

The Rossler system can be expressed as:

(14) $$\begin{array}{c} \dot{x}=-y-z\\ \dot{y}=x+ay\\ \dot{z}=xz-cz+b \end{array},$$

where *a* = 0.2, *b* = 0.2, and *c* = 5.7.

The Duffing system can be expressed as:

(15) $$\begin{array}{c} \dot{x}=y\\ \dot{y}=-bx+ay-y^{3} \end{array},$$

where *a* = 0.82 and *b* = −0.5.

The Chen system can be expressed as:

(16) $$\begin{array}{c} \dot{x}=a\left(y-x\right)\\ \dot{y}=\left(c-a\right)x-xz+cy\\ \dot{z}=xy-bz \end{array},$$

where *a* = 35, *b* = 3, and *c* = 28.

**Figure 4:**
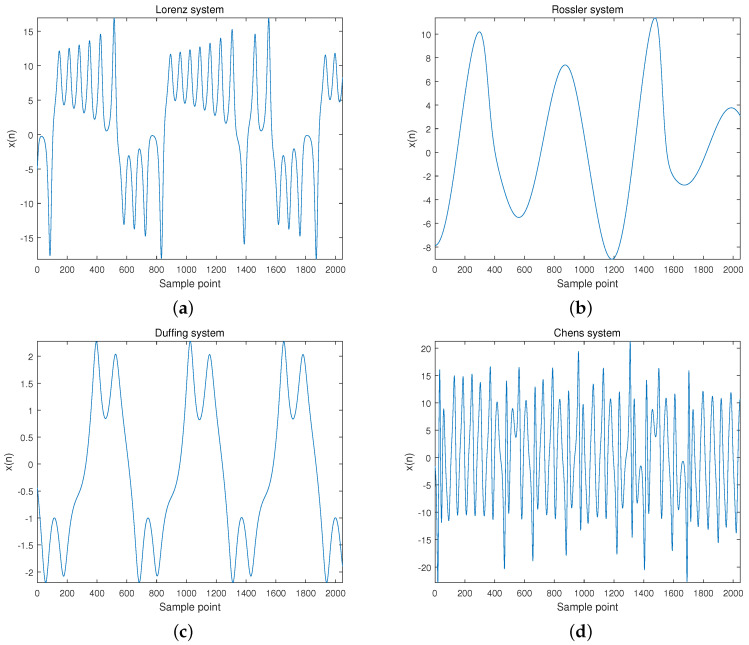
Timing diagrams of (**a**) Lorenz; (**b**) Rossler; (**c**) Duffing; and (**d**) Chen.

The random number generator was used to set the initial values of each chaotic system, and these equations were integrated by using a fourth-order Runge–Kutta method with a fixed step size of 0.01. The *X* component signal with a length of 2048 points was selected as the chaotic signal. The time-domain waveforms of Lorenz, Rossler, Duffing, and Chen are shown in Figure 4.

**Figure 5:**
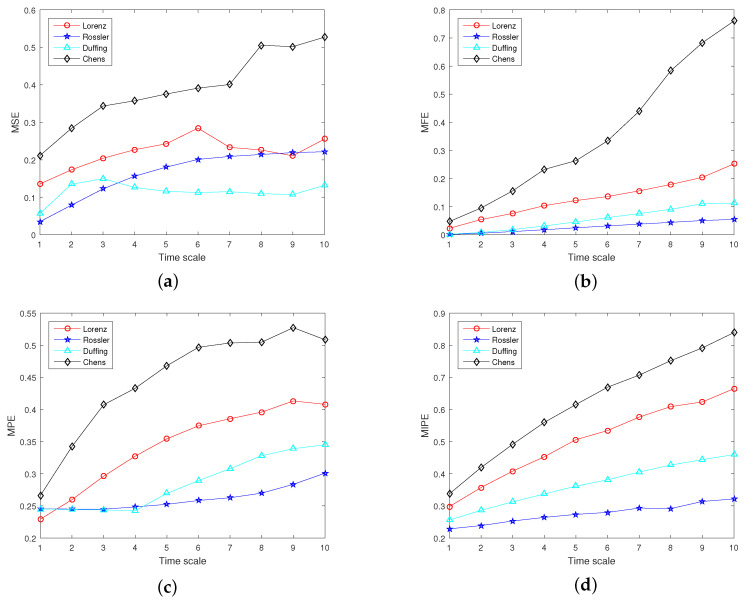
Entropy analysis results of chaotic systems: (**a**) multiscale sample entropy (MSE); (**b**) multiscale fuzzy entropy (MFE); (**c**) multiscale replacement entropy (MPE); and (**d**) multiscale improved permutation entropy (MIPE).

For comparison, the multiscale sample entropy (MSE) [49], multiscale fuzzy entropy (MFE) [53], multiscale replacement entropy (MPE) [54] and MIPE were selected to analyze these four chaotic systems. According to [48], the parameters were set as follows: the embedding dimension m=4, the time delay t=1, the discrete level L=4, and the time scale s=10. The following studies used the same parameters. The analysis results are shown in Figure 5. The entropy results tended to increase with the increase of the time scale, which is reasonable. With the increase of the coarse-graining process, the change in the unit data length rose, increasing the data randomness. The comparison in Figure 5 shows that the MSE is difficult to distinguish the Lorenz, Rossler, and Duffing systems. For a small time scale, MFE and MPE cannot distinguish the Rossler system from Duffing chaotic systems, while MIPE can distinguish all chaotic signals. MIPE has a strong ability to classify multiscale complexity signals. Therefore, MIPE was utilized to extract the entropy characteristics of running power equipment sound in this paper.

## 4. Feature Extraction of Power Equipment Sound

**Figure 6:**
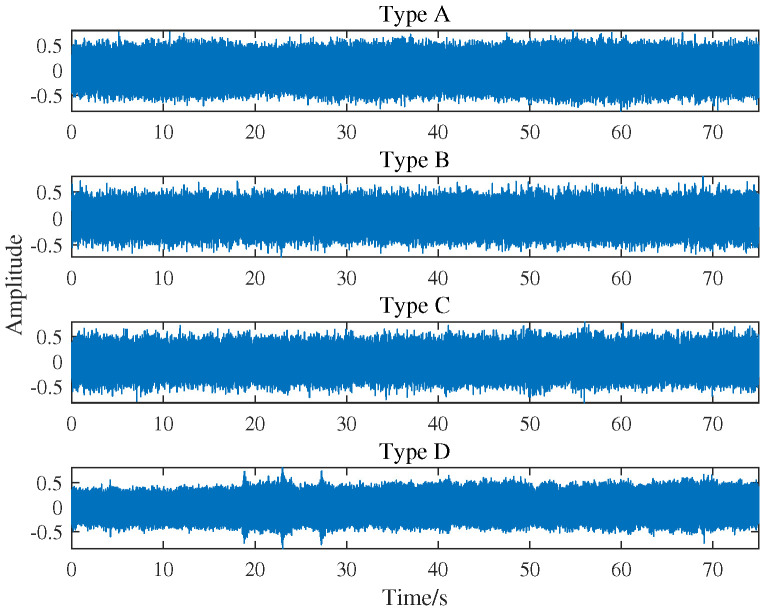
Sound waveform of four types of running power equipment.

In this section, the proposed feature extraction algorithm is used to analyze the sound of actual power equipment. The audio data used in this paper came from four types of key power equipment measured in a power plant under the same conditions. Type A, B, C, and D respectively represent the feeder connecting shaft, induced fan blade, coal grinder, and circulating water pump. For each type of electrical equipment, we acquired 50 samples of 1.5 s duration. The sampling frequency of each sample is 16 kHz. The sound waveforms of four power equipment are shown in Figure 6.

### 4.1. Feature Extraction Based on ICEEMDAN and MIPE

**Figure 7:**
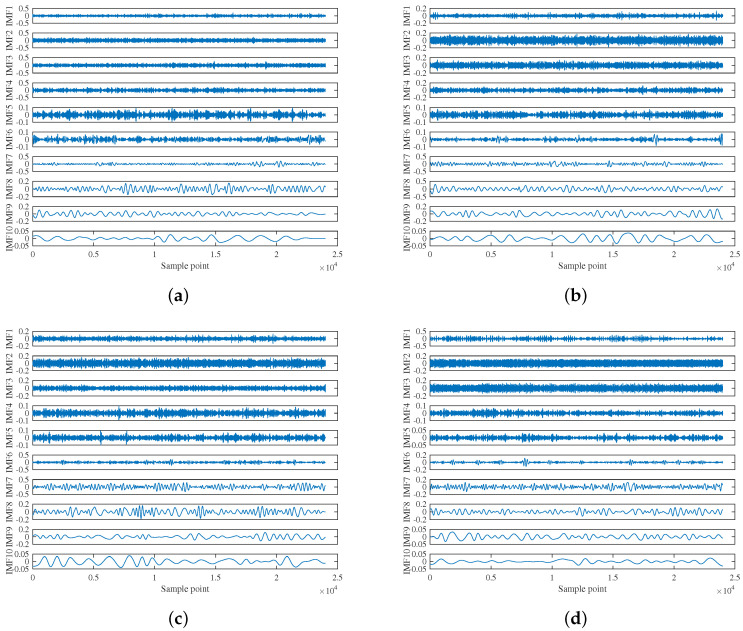
ICEEMDAN analysis results of four types: (**a**) Type A; (**b**) Type B; (**c**) Type C; and (**d**) Type D.

**Figure 8:**
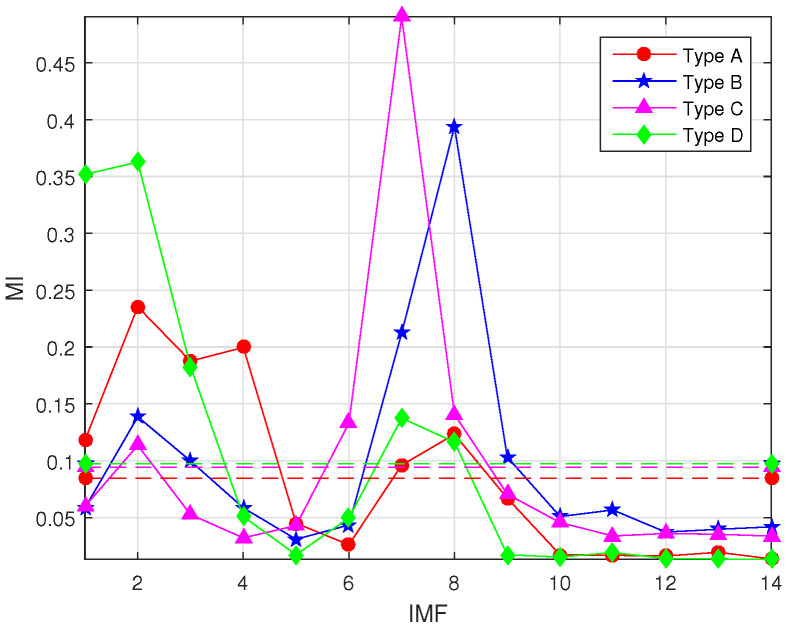
MI and meanMI of four types of sound.

The effectiveness of the proposed feature extraction method is verified and demonstrated in this subsection. As shown in Figure 7, ICEEMDAN was used to decompose randomly selected experimental data to produce a group of IMFs with different center frequencies. For clarity, only the first ten IMFs are demonstrated. Figure 8 shows the MI of each IMF and the original signal, where the dashed line of the corresponding color represents the corresponding meanMI to distinguish the signal IMFs.

**Figure 9:**
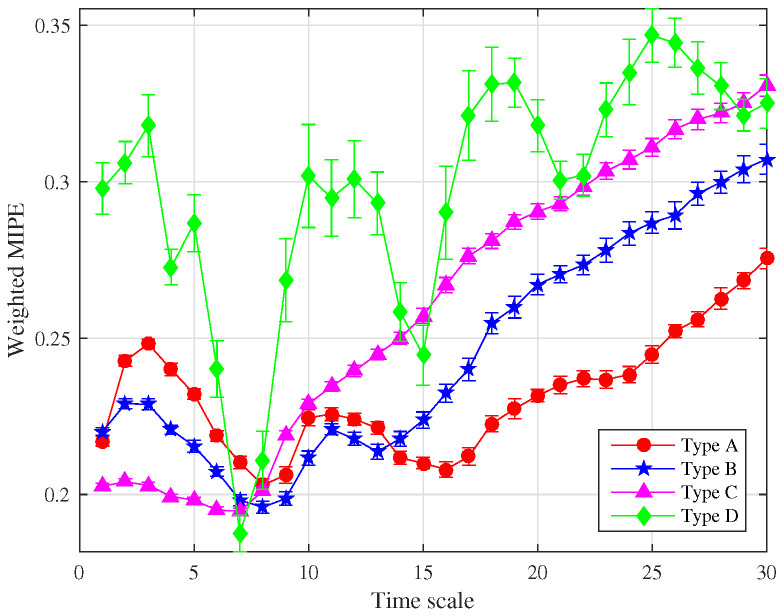
Weighted MIPE results.

After eliminating the noise IMFs, the norMI and MIPE of each signal IMFs were calculated. Then norMI was used to weight the corresponding MIPE results. The weighted MIPE results took into account the importance of each signal IMFs. The multiscale MIPE results of the four power devices obtained through an average of 50 sets of experimental data are shown in Figure 9. The scale factor was selected as *s* = 1–30, and the error bars represent the SD of the weighted MIPE values.

**Figure 10:**
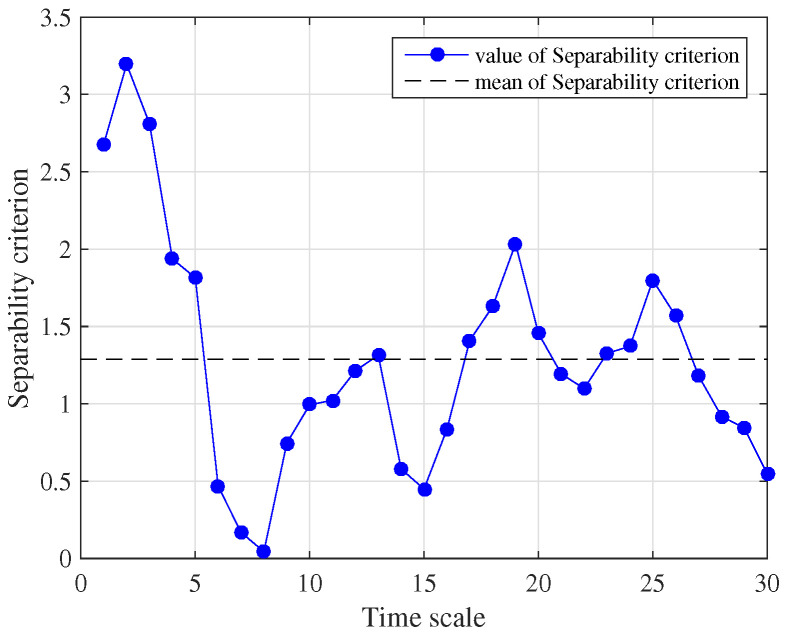
Distribution of separable criterion.

The separability criterion was used to analyze each dimension of the weighted MIPE results. The distribution of SC is shown in Figure 10, where the black dashed line represents the mean value of 30 SCs. For the separability criterion larger than the mean of separability criterion value, the feature can be defined as an effective one. Comparing Figure 9 with Figure 10, it can be seen that the separability criterion distribution had a positive correlation with the dispersion degree of the weighted MIPE result. The SC was large when these four types of weighted MIPE results were significantly different. Hence, it is obvious that the dimension reduction extraction based on the separability criterion is effective.

**Figure 11:**
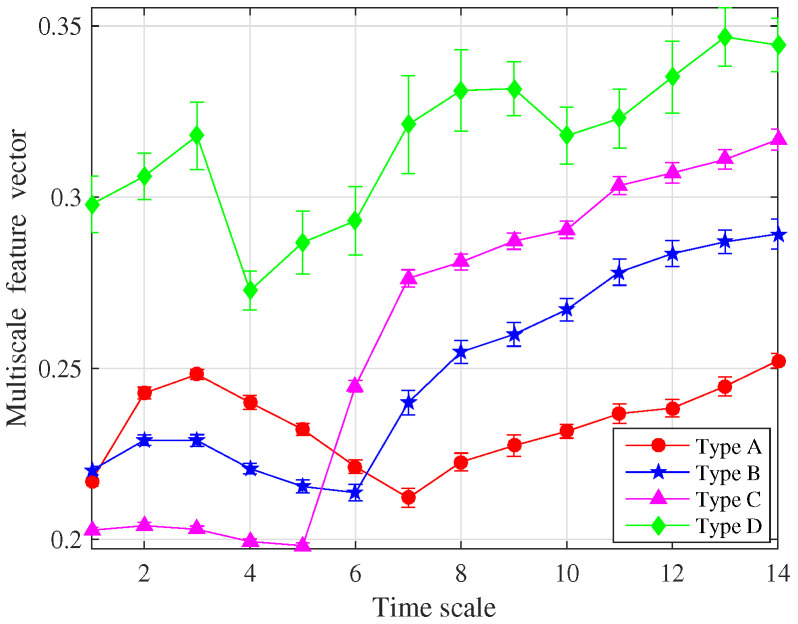
The multiscale entropy feature vector of four types of running power equipment sound.

The multiscale entropy feature vector was obtained after the feature dimension reduction, as shown in Figure 11. For different types of running power equipment sound, the entropy feature vectors are clearly different, indicating that the extracted features are effective for power equipment classification. Moreover, the error bar on each scale factor is low, proving the reliability of the proposed method.

**Figure 12:**
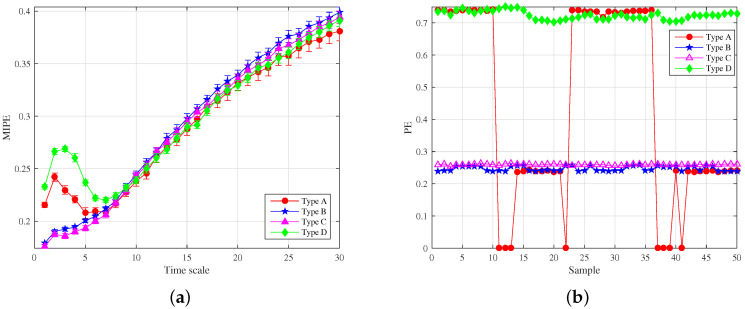
Analysis results of other feature extraction methods: (**a**) MIPE; and (**b**) CEEMDAN-PE.

For comparison, the same experimental data were analyzed using the feature extraction method in [48,55]. The feature extraction results of MIPE [48] are shown in Figure 12a, where the mean value of IPE and its standard deviation error is drawn. The IPE values of type B and C overlap in contrast with that in Figure 11. In particular, the IPE values of the four types of signals all overlap and are difficult to distinguish when s>6. The reason lies in the lack of noise elimination before the MIPE calculation, and the results are influenced by the noise, which becomes larger with the coarse-graining process. The analysis results of CEEMDAN-PE [55] are demonstrated in Figure 12b, where the abscissa represents the different types of samples and the ordinate represents the PE value. The parameters of the algorithm were set as same as those in [55]. Because CEEMDAN-PE was based on a single scale, the relationship between entropy and time scale was discarded. It can be seen from Figure 12b that the PE values of type B and type C are close to each other. The results of type A overlap with those of other types, making them difficult to be distinguished.

**Figure 13:**
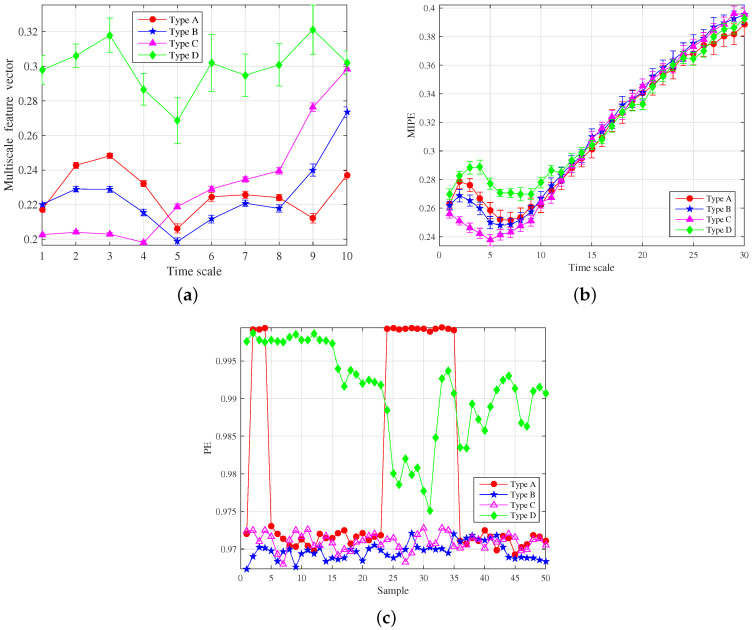
Feature extraction results under 0 dB condition: (**a**) the proposed method; (**b**) MIPE; and (**c**) CEEMDAN-permutation entropy (PE).

To study the effect of the feature extraction method above under a noisy condition, the white Gaussian noise was added into the running power equipment sound to produce different SNR conditions. As shown in Figure 13, the methods mentioned above were applied to extract features for the sound signal under the condition of 0 dB. Compared with the effect shown in Figure 11 and Figure 12, CEEMDAN-PE, which is based on a single scale, becomes invalid due to noise interference. The results of type A, type B, and type C overlap more, greatly increasing the difficulty and accuracy of classification. Similarly, due to the lack of effective de-noising process, the performance of MIPE declined rapidly, and the differentiation of MIPE of each type decreased. Especially when *s* = 1–5, SD of the sample data increased significantly, which reduces the robustness of the classification. On the contrary, the added noise has little influence on results obtained by the proposed method, which proves its credibility.

### 4.2. Classification of Power Equipment

In order to verify the feature extraction method, a widely applied classifier—support vector machine (SVM)—was used to process the features extracted in Section 4.1. For each type of power equipment, 25 zero noise samples were randomly selected for training and the remaining 25 samples were used for validation. We repeated this ten times, and took the average of the results as the classification results, as listed in Table 1. To study the role of the separability criterion in the proposed algorithm, the classification accuracy of the weighted MIPE feature vectors without feature dimensionality reduction was calculated. As shown in Table 1, the classification accuracy of the weighted MIPE feature vector and the proposed method was higher than other feature extraction methods, whether the noise was added or not. Moreover, the separability criterion can significantly improve the classification accuracy and enhance the robustness of the algorithm under serious-noise conditions.

As can be seen from Table 1, the classification accuracy is in good agreement with the feature extraction results in Section 4.1. For a clean signal, the proposed method achieved an accuracy rate of 96.7%, 12.1%, and 27.8% higher than that of MIPE and CEEMDAN-PE, respectively. The SNR decreasing to 5 dB, the classification accuracy of the proposed method declined to 89.9%, while the classification accuracy of MIPE and CEEMDAN-PE dropped to 52.2% and 65.8%, respectively. As the SNR dropped to 0 dB, the classification accuracy of MIPE and CEEMDAN-PE algorithms dropped to 46.6% and 64.9% respectively. In contrast, the proposed method still had a classification accuracy of 92.7%.

Comparing the classification results of MIPE and CEEMDAN-PE, the MIPE performance declined rapidly under high noise conditions due to the lack of the de-noising process. Similarly, for the classification rate of a clean signal, the effective entropy algorithm helps to extract the nonlinear features in the signal and significantly improves the classification accuracy. The results above prove the validity of the algorithm from another angle.

## 5. Conclusions

A separability criterion is proposed to extract effective features from the sound of running power equipment. Compared with the existing feature extraction technology, it is based on multiple scales and removes noise before calculating MIPE. Moreover, the proposed method uses norMI to weight MIPE results, which takes into account the importance of signal IMFs and utilizes the separability criterion to extract effective features and reduce dimensions of the weighted MIPE results. In this way, the robustness and accuracy of the equipment classification are improved significantly. The validation of the proposed algorithm is proved by analyzing acoustics signals measured from four types of electrical equipment, which demonstrates more precise identifications and higher sensitivity than the MIPE and CEEMDAN-PE. Therefore, the proposed method is more reliable and suitable for the entropy feature extraction for the running power equipment sound in practice, and it provides a supplement to monitoring techniques for electrical equipment. 

## Figures and Tables

**Table 1:** Classification accuracy for four types of power equipment by support vector machine (SVM).

Method	Clean Signal	5 dB	0 dB
The proposed method	**96.7%**	**89.9%**	**92.7%**
Weighted MIPE	93.8%	81.3%	67.2%
MIPE [48]	84.6%	52.2%	46.6%
CEEMDAN-PE [55]	68.9%	65.8%	64.9%

## Abbreviations

The following abbreviations are used in this manuscript:

EMD	Empirical Mode Decomposition
IMF	Intrinsic Mode Function
EEMD	Ensemble Empirical Mode Decomposition
CEEMDAN	Complementary Ensemble Empirical Mode Decomposition with Ddaptive Noise
ICEEMDAN	Improved Complementary Ensemble Empirical Mode Decomposition with Ddaptive Noise
SE	Sample Entropy
FE	Fuzzy Entropy
PE	Permutation Entropy
IPE	Improved Permutation Entropy
UQ	Uniform Quantification
MIPE	Multiscale Improved Permutation Entropy
norMI	Normalized Mutual Information
SNR	Signal-to-Noise Ratio
SD	Standard Deviation
MI	Mutual Information
meanMI	Mean Mutual Information
SC	Separable Criterion Value
MSE	Multiscale Sample Entropy
MFE	Multiscale Fuzzy Entropy
MPE	Multiscale Permutation Entropy
SVM	Support Vector Machine
